# Differential expression of metallothioneins (MTs) 1, 2, and 3 in response to zinc treatment in human prostate normal and malignant cells and tissues

**DOI:** 10.1186/1476-4598-7-7

**Published:** 2008-01-21

**Authors:** Hua Wei, Mohamed Mokhtar Desouki, Shufei Lin, Dakai Xiao, Renty B Franklin, Pei Feng

**Affiliations:** 1 Department of Biomedical Sciences, Dental School, University of Maryland at Baltimore, Maryland, USA; 2 Department of Pathology and Lab of Medicine, Medical University of South Carolina, Charleston, South Carolina, USA; 3 Greenebaum Cancer Center, University of Maryland at Baltimore, Baltimore, Maryland, USA

## Abstract

**Background:**

The disturbance of zinc homeostasis featured with a significant decrease of cellular zinc level was well documented to associate with the development and progression of human prostate malignancy. We have previously reported that zinc treatment induces prostate malignant cell apoptosis through mitochondrial pathway. Metallothionein (MT) is a major receptor/donor of zinc in the cells. However, the studies on the expression of MT in association with the prostate pathological and malignant status are very limited, and the zinc regulation of MT isoform expression in prostate cells remains elusive. The goals of this study were to define the expression of endogenous MTs, the isoforms of MT 1, 2, 3 at both messenger ribonucleic acid (mRNA) and protein levels; and to investigate the zinc effect on MT expression in normal prostate, benign prostatic hyperplasia (BPH) and malignant PC-3 cells, and in relevant human tissues. Cellular MT proteins were detected by immunohistochemistry, fluorescence staining and Western blot analysis; reverse transcription polymerase chain reaction (RT-PCR) was used to determine the MT isoform-specific mRNAs.

**Results:**

Our results demonstrated a significant suppression of endogenous levels of MT1/2 in malignant PC-3 cells (95% reduction compared to the normal prostate cells) and in human adenocarcinoma tissues (73% MT1/2 negative). A moderate reduction of MT1/2 expression was observed in BPH. Zinc treatment remarkably induced MT1/2 expression in PC-3 and BPH cells, which was accordant with the restored cellular zinc level. MT 3, as a growth inhibitory factor, was detected and up-regulated by zinc mainly in BPH cells.

**Conclusion:**

This study provided evidence of the association of attenuated MT1/2 with prostate tumor progression, and the zinc induction of MT1/2 expression resulting in cellular zinc restoration. The results suggest the potential of MT1/2 as a candidate biomarker for prostate cancer and the utilization of zinc in prostate cancer prevention and treatment.

## Background

Zinc is an essential element involved in many cellular functions and is required by approximately 300 enzymes for their biological activities [1]. In humans, deficiency of zinc might inhibit growth [2], but it is also closely related to increased risk of certain malignant tumors [3,4]. Normal prostate contains the highest zinc level, but a dramatic decrease of cellular zinc (60–70% loss) was found in malignant prostate cells [5]. We have demonstrated that zinc exposure induces apoptosis in malignant prostate PC-3 and benign hyperplasia prostate (BPH) cells, but not in normal prostate HPR-1 cells [6,7]. Despite few studies on the relationship of zinc accumulation and zinc-induced prostatic cell apoptosis [8-10], the mechanisms of the disturbance of zinc homeostasis and zinc restoration in relation to pathogenesis and malignancy of prostate tissues remain unclear.

Cellular zinc homeostasis is modulated by many factors such as zinc transporters (ZnTs and ZIPs) and metallothioneins (MTs), which are involved in the aspects of zinc transport, trafficking, and signals [11]. Among these factors, MTs are of low molecular weight (~6 kd); molecules and the cysteine-rich motifs in α and β domain are responsible for their zinc-binding property, hence, the Zn-MT-thionein conjugated pair functions as a receptor/donor for other zinc-related proteins [12]. MTs are ubiquitously expressed in most cells and tissues and play important roles in many biological processes such as metal ion homeostasis and detoxification, protection of cells from the damage caused by oxidative stress, cell proliferation and apoptosis, and in some aspects of the carcinogenic process [13,14].

MT genes belong to a super family with characteristics common to equine MT, first isolated half a century ago [15,16]. Since then, four isoforms (MTs 1, 2, 3, and 4) were identified [17]; among them, MTs 1 and 2 are the major isoforms expressed in most adult mammalian tissues. MT 3 was originally found exclusively in the normal human brain as growth inhibitor factor [18], and lately, the expression of MT 3 was further identified in kidney, breast, pancreas, intestine, bladder, and prostate cancer [19,20]. MT 4 expression was reported in the stratified squamous epithelium and has an important role in cell differentiation [21].

The zinc regulation of MT gene transcription was through metal response elements (MREs), which are present in multiple copies within the proximal promoters of MT genes [22]. MREs seem to be MT isoform-dependant and cell-type specific. In prostate cells, high concentration of zinc (100 μM)-induced MT1/2 expression was studied in malignant PC-3 cells [13,23]. However, the information regarding zinc regulation of MT1/2 in normal and BPH cells is very limited, whereas contradictory observations of this in human prostate tissues were reported. In addition to MT1/2, several MREs on the promoter region of MT 3 gene were also identified; however, the effect of zinc on MT 3 expression has not been clarified [24]. To date, questions of whether MT 3 is expressed in human prostate and whether such expression is regulated by zinc associated with its growth-inhibitory role remain unclear and need to be studied.

In addition to the previous findings from different investigations, this study for the first time provided new evidence on zinc regulation of MT gene expression and elucidated the relationship between the gene expression and the cellular zinc homeostasis in relation to the pathogenesis status of the prostate tissues.

## Results

### Differential expression of endogenous MT1/2 in normal, benign hyperplasia, and malignant prostate cells correlates to the cellular zinc levels

**Figure 1:**
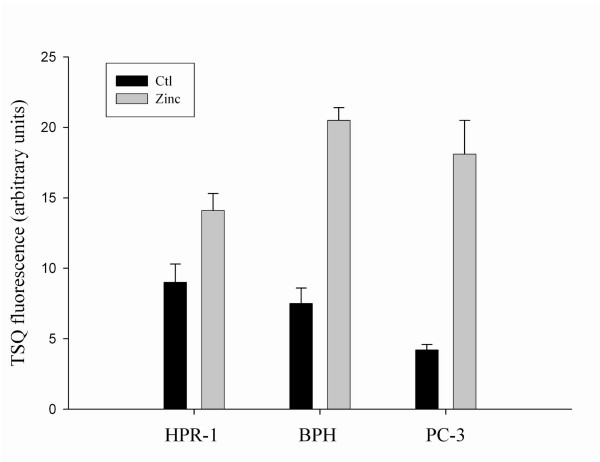
**Cellular zinc accumulation in human prostate cells**. Human prostatic HPR-1, BPH, and PC-3 cells were treated with (grey bars) or without (black bars) zinc (15 μM) in serum/pituitary extracts-free medium for 3 h. Thirty microliters of each cellular sample (200 mg of protein) were placed in a 96-well plate and the fluorescence of zinc labeled by TSQ was detected with a Microplate Reader (Fluoroskan Ascent, Labsystems).

**Figure 2:**
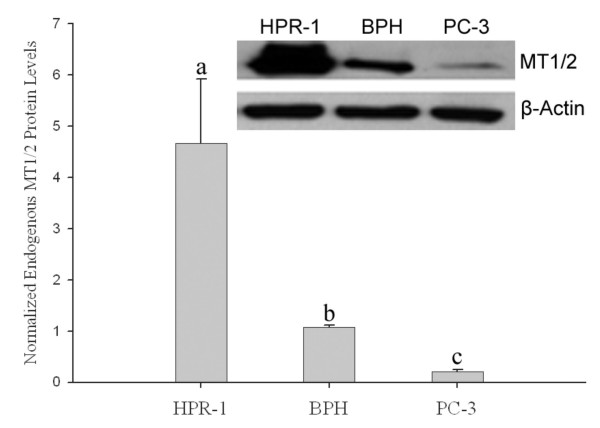
**Detection of endogenous level of MT1/2 proteins in human HPR-1, BPH, and PC-3 cells**. The protein extracts (30 μg) of each cell line were separated by SDS-PAGE, and cellular MT1/2 was detected by Western blot analysis with rabbit anti-MT1/2 antibody, and β-actin was used as an internal control (top panel). The density of specific signals of MT1/2 was analyzed and data are presented in lower panel. The different letters (a, b, c) represent the statistical significance (p < 0.05).

To ascertain the relationship between zinc homeostasis and MT1/2 gene expression in prostate tumorigenesis, the cellular zinc accumulation and the endogenous MT1/2 protein levels were detected in normal HPR-1, BPH, and malignant PC-3 cells. As shown in Fig. 1, higher endogenous cellular zinc was found in HPR-1 cells followed by BPH, and the lowest zinc level was detected in PC-3 cells. After the zinc treatment, a significant gaining of the cellular zinc was observed in both BPH and PC-3 cells with 1.7-fold and 3.3-fold increase, respectively. In contrast, the zinc level in HPR-1 cells was modestly enhanced (<0.6-fold). The cellular endogenous MT1/2 proteins in three cell lines were further evaluated by Western blot analyses (Fig. 2). The results showed that the highest endogenous level of MT1/2 was detected in HPR-1 cells, while PC-3 cells appeared to have the lowest endogenous MT1/2, only about 5% of that found in HPR-1. The MT1/2 in BPH cells was higher than in PC-3 cells, but still much less, about 25% of the amount found in HPR-1 cells. Apparently, MT1/2 in HPR-1 and BPH is significantly higher than in PC-3 cells; however, no significant difference of MT 1/2 between BPH and PC-3 cells was detected.

**Figure 3:**
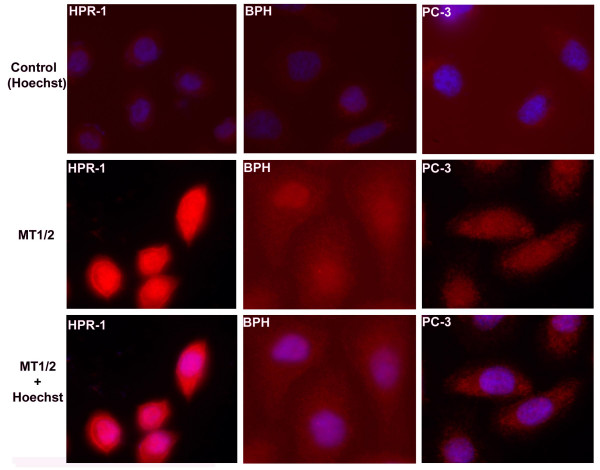
**Immunofluorescence detection of MT1/2 in HPR-1, BPH, and PC-3 cells**. The immunofluorescence statining of MT1/2 was examined in the cells, HPR-1 (left), BPH (middle), and PC-3 (right). The background of non-specific staining was monitored using the red FLURO-conjugated secondary antibody only indicated as the control (top row), in which the cells were also stained with Hoechost 33258 shown the blue-stained nuclei to remark the cells. The differential immunoreactivities of the cells to MT1/2 antibody were determined by the intensity of red fluorescence between the cell lines (middle row). The images with both MT1/2 and Hoechost nuclear staining (bottom row) were the attempts to detect the cellular distribution of MT1/2. The pinkish colour resulted from the merged blue and red colour was observed in some nuclei of HPR-1 cells. All images were observed and recorded under the same settings of a fluorescence microscope with the magnification of × 600.

The cellular distribution of MT 1/2 was studied by using immunofluorescence staining (IFS) shown in Fig. 3. A strong fluorescence intensity of MT1/2 signals was identified in HPR-1 cells, while a much weaker immunoreactivity was observed in BPH and PC-3 cells. From the data of IFS it was further evident that MT1/2 expression displayed a cell-type specific pattern in the prostate cells. Although the cytoplasmic and nuclear localization of MT1/2 was observed in all three cell lines, a higher ratio of nuclear MT1/2 versus cytoplasmic distribution appeared only in HPR-1 cells. The specificity of the MT1/2 signals was confirmed in the negative controls in which only the secondary antibody was applied (top panels of Fig. 3).

### The zinc induction of MT1/2 expression is cell-type specific in prostate cells

**Figure 4:**
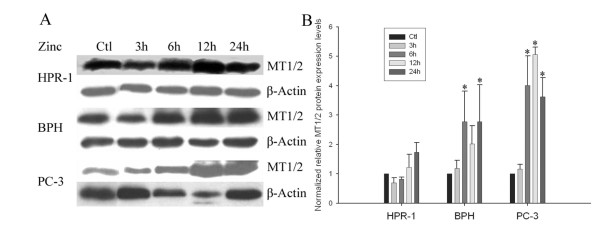
**Regulation of MT1/2 protein expression in HPR-1 BPH, and PC-3 cells**. **A**. The cells were treated with zinc (15 μM) for 3, 6, 12, and 24 h, respectively, after 24 h serum-depletion. The protein extracts (30 μg) of each cell line were separated by SDS-PAGE, and human MT1/2 was detected by Western blot analysis with rabbit anti-MT1/2 antibody, and β-actin was used as an internal control. **B**. The density of specific bands was analyzed and data are presented.

As we reported above, the levels of cellular zinc were correlated to the endogenous MT1/2 expression, and zinc restoration was higher in BPH and PC-3 cells than in HPR-1 cells (Fig. 1). To extend this important relationship, the effect of zinc on the induction of MT1/2 was investigated and a time-course study of zinc-induced MT1/2 was conducted with three cell lines (Fig. 4). The results of Western blot analyses revealed that zinc exposure (1 μg/ml) of BPH and PC-3 cells resulted in a significant increase in the MT1/2 determined early, at 6 h, by about 2.8-fold and 4-fold compared to the controls, respectively. This distinct induction effect of zinc on MT1/2 was continuously observed up to 24 h in both BPH and PC-3 cells. In contrast, in HPR-1 cells only a slight increase of MT1/2 expression, with no significant difference to the controls, was detected after 12 h of zinc treatment. The results demonstrated a dynamic association of cellular zinc and MT1/2 expression with a cell-type specific property in prostate cells.

### Effect of zinc on the expression of MT 3 mRNA and protein levels in prostate cells

**Figure 5:**
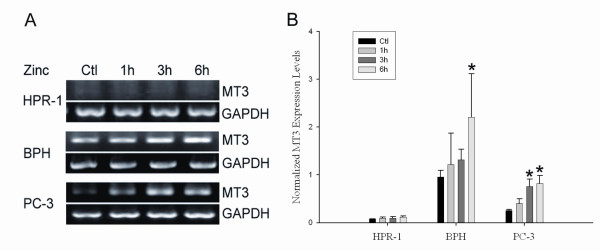
**Detection of zinc regulation of MT3 gene expression in HPR-1, BPH, and PC-3 cells by RT-PCR**. **A**. Cells were treated with or without zinc (20 μM) following 24 h serum-depletion. Zinc effect on MT 3 mRNA levels was evaluated at 1, 3, and 6 h of zinc treatment. Total RNA (0.2 μg) was used for 25 μL RT-PCR reaction. The sequences of MT 3 primers are: 5'*CCGTTCACCGCCTCCAG*3' (upper); 5'*CACCAGCCACACTTCACCACA*3' (lower). GAPDH was used as an internal control to normalize MT-3 gene expression. **B**. The density of specific bands was analyzed and data are presented.

To date, the zinc effect on the transcriptional and translational level of MT 3 in prostate cells has not yet been reported. In order to further determine whether zinc regulated MT 3 gene expression, a time-course study of zinc effect on MT 3 gene transcription was conducted by using RT-PCR (Fig. 5). The results showed that among three cell lines, the highest endogenous level of MT 3 mRNA was identified in BPH cells while a remarkably lower level, about 25% of that in BPH cells, was detected in PC-3 cells; and there was only a marginally detectable amount found in HPR-1 cells (Fig. 5). Most interestingly, zinc treatment significantly increased MT 3 mRNA in BPH (2-fold) and PC-3 (3-fold) cells at late time points, at 6 h and 3–6 h, respectively; however, no zinc induction of MT 3 mRNA was found in HPR-1 cells.

**Figure 6:**
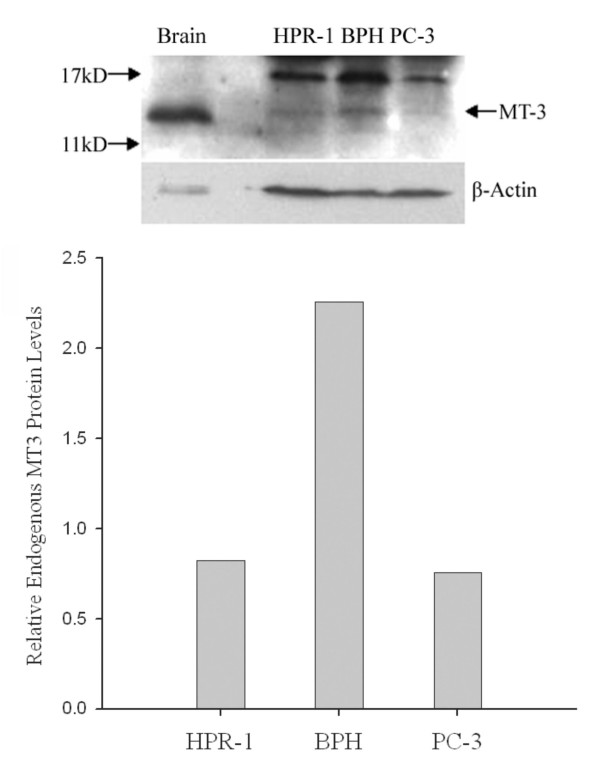
**Endogenous level of MT 3 protein in HPR-1, BPH, and PC-3 cells**. The cells were grown to confluence and harvested. The protein extracts (50 μg) of each cell line were separated by SDS-PAGE, and the MT 3 was detected by Western blot analysis with rabbit anti-MT 3 antibody, and β-actin was used as an internal control (Top panel). The density of specific MT 3 signals was analyzed as described in the Materials and Methods, and the data are shown on the lower panel. The experiment was repeated at least thrice.

**Figure 7:**
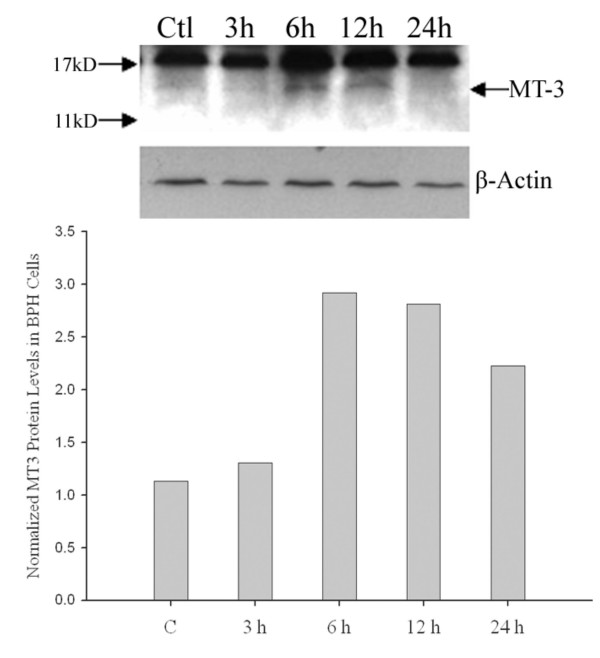
**Detection of zinc regulation of MT 3 expression in BPH cells by Western blot analysis**. BPH cells were treated with zinc (15 μM) or without zinc (control) for 3, 6, 12, and 24 h, respectively. The protein extracts (50 μg) of samples from each time point were separated by SDS-PAGE, and the MT 3 was detected by Western blot analysis (top panel) with rabbit anti-MT 3 antibody and β-actin was used as an internal control. The density of specific MT 3 signals was analyzed and data are shown on the lower panel. The experiment was repeated at least thrice.

We further investigated whether MT 3 is expressed at a functional level and induced by zinc similar to that of the MT1/2 isoforms (Fig. 4) in prostate cells. Firstly, the endogenous level of MT 3 protein in three cell lines was examined, and the signal detected by a specific polyclonal antibody against MT 3 was only identified in BPH cells compared to the positive control of the human brain tissue, in which endogenous MT 3 protein is highly expressed (Fig. 6). In contrast to the MT 3 mRNA levels obtained from RT-PCR assays, the MT 3 protein was neither detectable in PC-3 nor in HPR-1 cells. Next, we conducted a time-course study of zinc (15 μM) effect on MT 3 in BPH cells by Western blot analyses (Fig. 7). The expression of MT 3 displayed a zinc-response pattern, and the zinc induction of MT 3 was observed in BPH cells with about 3-fold increases after 6 – 12 h of zinc treatment (Fig. 7).

### Detection of MT1/2 expression in different human prostate tissues by using immunohistochemistry (IHC)

**Figure 8:**
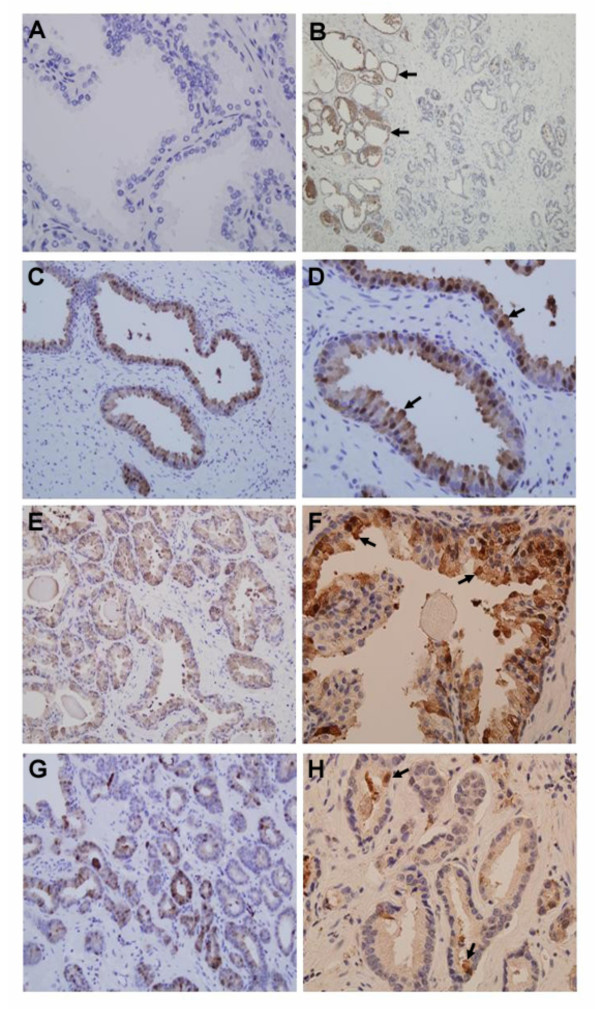
**Immunohistochemistry staining of MT 1/2 in human prostate tissues**. Normal (C &D) and BPH (E & F) prostate tissues demonstrate MT 1/2 immunoreactivity with positive and/or patchy intensity in the glandular epithelial cells. The malignant tissues (G & H) appear to be negative to MT 1/2 staining in most of the cells with a few scattered stained cells. Control (A) was stained without primary MT 1/2 antibody. Immunoreactivity in a mixed tissue (B) of malignancy and BPH is indicated by arrows. Magnifications: 200-fold (B, C, E, G) and 400-fold (A, D, F, H)

MT1/2 expression in human prostatic tissues obtained from patients with normal, BPH and/or prostatic adenocarcinomas was evaluated by using IHC method. The specificity of MT1/2 signals identified by IHC staining was determined by using the negative controls in which only secondary antibody was applied resulting in no MT1/2 immunoreactive signals (Fig. 8A). The results showed that various immunoreactivities for MT1/2 were detected in differential prostate tissues, and the immunoreactivity was mostly confined to the epithelial cells of prostatic glands (Fig. 8). In contrast, the stroma cells showed negative-to-weak immunoreactivity for MT1/2, and the connective tissues appeared to have no reaction to the MT1/2 antibody. In normal prostate tissue (Fig. 8C & D), the distinct higher intensity of immunostaining was observed in the nuclei, while the cytoplasm showed an evenly distributed staining in almost all glandular epithelial cells. The results showed that in BPH tissue (Fig. 8E &8F) a patchy MT1/2 staining of epithelial cells was observed, that is, lacking uniformity of immunoreactivities resulting in uneven immunostaining intensity among the cells. In general, the majority of the epithelial cells in BPH tissue remained immunoreactive to MT1/2, while in some cells a stronger reaction had occurred than in other cells. A significant reduction of the immunostaining intensity was observed in the malignant adenocarcinomatous glands (Fig. 8G &8H). Only a few malignant cells appeared to have had the immunoreaction for MT1/2, whereas the nuclei and cytoplasm showed a negative reaction to MT1/2 in most of the malignant cells. A clear evidence of the cell-type specificity of MT inmmunoreactivity is shown in Fig. 8B, in which no MT1/2 staining was detected in the field of malignant prostate tissue, while it was positively detected in adjacent defined BPH tissues in the same field.

A summary of the MT1/2 IHC scores in prostatic adenocarcinomas and associated defined BPH tissues collected from 23 patients is presented in Table 1. The results of the semi-quantitative scoring showed that the positive MT1/2 immunostaining was found in all BPH tissues, and about 87% of examined BPH tissues appeared to have strong immunoreactivity for MT1/2 (Scores: ++ & +++). In contrast, the negative immunoreactivity for MT1/2 was determined in most prostatic adenocarcinomatous foci (73% negative) shown as 13 negative over a total 18 of examined cases.

**Table 1:** Summary of the immunorectivity to MT 1/2 in human BPH and prostate adenocarcinoma tissues


Case no.	Grade*	MT1/2 IHC score^a^**	
		
		BPH^b^	Prostatic carcinoma

1	3	++	Negative
2	3	++	Negative
3	1	++	Negative
4	2	++	+
5	3	++	Negative
6	N/A	+++	N/A
7	2	++	Negative
8	N/A	++	N/A
9	1	++	Negative
10	3	++	Negative
11	2	++	Negative
12	3	++	Negative
13	2	++	Negative
14	2	++	Negative
15	3	+	Negative
16	N/A	++	N/A
17	N/A	++	N/A
18	2	++	++
19	2	++	+
20	N/A	+	N/A
21	3	++	Negative
22	2	++	++
23	3	+	++

Slides were prepared from formalin fixed paraffin embedded tissues.

* Gleason grade evaluated using WHO standard [41]

^a^** Semi-quantitative scoring of immunoreactivity was done as follows [40]:

Negative, no positive cells; score +, <10% positive cells; score ++, 10–50% positive cells; score +++, > 50% positive cells

## Discussion

The findings described above allow us to define the relationship between the down-regulated MT expression and the decreased endogenous level of zinc in human prostate malignant cells and tissues. The disturbance of zinc homeostasis in prostate would significantly impair the cellular metabolism and growth as discussed previously [5,6]. Now, a critical issue could be raised: Does the low level of cellular zinc lead responsively to a suppressed endogenous MT level or does the down-regulated MT expression result in a significant depletion of cellular zinc underlying the tumorigenesis of prostate tissues. Most recently, an important study on the mechanism of MT gene regulation by metal response element-binding transcription factor-1 (MTF-1) was reported by Dr. R. Tjian's group [25]. In this study, the robust response of the MT genes to metal was used as a model; MTF-1, a core facilitating factor, and two central regulatory factors, transcription factor II D (TFIID) and mediator (MED), were identified as major players in modulating the transcriptional response of MT to metal. The MTF-1 recruits TFIID, which then binds MT promoter DNA and subsequently activates transcription upon recruitment of the MED [26]. The appropriate expression of the endogenous MT genes is achieved only when these coactivators are balanced. Intriguingly, they found that the MTF-1 requires different coactivator subunits depending on the context of the core promoter (the stability of the multi-subunit coactivator complexes can be compromised by loss of a single subunit), leading to the potential control of fine-tune transcriptional regulation. We observed that the endogenous level of MT in the prostate cells is cell-type specific and that the oncological trend of the endogenous MT may be closely related to MTF-1-involved MT gene regulation.

The alteration of MT1/2 gene expression was not only found in human prostate, but was also discovered in primary human hepatocellular carcinomas [27], in which the suppression of MT1/2 gene expression is mediated through phosphatidylinositol 3-kinase (PI3K) signaling pathway by the inactivation of the CCAAT/enhancer binding protein α (C/EBPα). On the other hand, MTF-1 interacted with C/EBPα as a complex and subsequently activated MT1/2 gene expression. An early study also indicated that in prostate cancer cell lines the MT1 promoter activity was suppressed by promoter methylation of cytosine within the CpG dinucleotide region [28]. Besides the MT promoter methylation, the possibility of dysfunctional interaction of MTF-1 with nuclear factor I (NFI) was also seriously considered [28]. These findings highlight insights for future investigations for the regulation of MT gene expression in association with the prostate zinc homeostasis and tumorigenesis.

MTs, as zinc donors/receptors, play a key role for zinc-related proteins and enzymes, which are involved in many crucial cellular functions including cell metabolism, signaling transduction pathways, and nuclear gene transcriptional regulation. Concordantly with the functions, the extensive cellular distribution of MT1/2 was observed in normal prostate HPR-1 cells with the intensity of immunofluorescence staining in the cytosol and even stronger signals in the nuclei (Fig. 3). However, a substantive decrease of MT1/2 intensity was displayed in both BPH and PC-3 cells, which confirmed the result of the Western blot analysis. In extension of previous observations of high IHC staining in the nuclei of BPH cells [29], we found that normal HPR-1 cells exhibit much more aggregation of MT1/2 in nuclei, than BPH and PC-3 cells do. This suggests that the suppression of MT expression and the alteration of MT distribution in BPH and PC-3 cells may negatively influence the cellular function.

The results of the suppressed MT1/2 expression in prostate malignant cells led us to further investigate MT1/2 expression in human prostatic tissues including the normal, BPH, and adenocarcinomas. At the present time, only few studies provided limited information of MT1/2 expression in human prostate tissues. Among these data, the correlation of the positive IHC staining of MT1/2 and the adenocarcinoma status was variable ranging from 33% (15/45) [30], 67% (20/30) [31] to 100% (5/5) [32]; while some reports indicated that the Gleason grades of the tumor tissue scoring from low to high reversely corresponded to the intensity of MT1/2 IHC staining [32,33].

In this study, normal and BPH tissues give images of intensive and/or patchy MT1/2 IHC staining, which are consistent with some previous observations [30,31,33]. Moreover, we determined the negative and/or weak MT1/2 IHC staining in the majority of adenocarcinomatous cells (72%), which is in agreement with one of most recent reports (67%) [31], while other studies showed higher staining signals of MT1/2 in prostate malignant tissues, 33% and 0%, respectively [32,33]. The discrepancy of the IHC staining data may result from the differential MT1/2 antibodies and the variable staining methods. Apparently, examining more prostate malignant tissues with a standard IHC method will provide significant information of MT1/2 expression, which could be considered as the candidate gene used for early diagnosis and gene therapy for prevention and targeting of prostate caner.

In addition to MT1/2, we have also determined, for the first time, the zinc regulation of MT 3 expression at both transcriptional and translational levels in three prostate cell models. Although the MT 3 was initially thought to be a neuron-specific MT isoform possessing a neuronal cell growth inhibitory activity [18,34], in recent years the demonstration of a wider, non-neural distribution of human MT 3 [35,36] motivated a study of MT 3 in prostate, in which a variable nature of MT 3 expression was detected in prostate tissues and cells [37]. Contrary to expectations based on the previous study [37], we found that among three cell models, only BPH cells profoundly express the highest endogenous level of MT 3, which was also significantly up-regulated by zinc at both mRNA and protein levels (Figs. 5 and 7). Also, neither normal HPR-1 nor malignant PC-3 cells seem to express essential amounts of MT 3 or to respond to zinc induction of the MT 3, thereby suggesting a cell-type specific nature of the MT 3 gene expression and regulation. The characteristic inhibitory effect of MT 3 on cell growth was discriminated by promoting cell necrosis from cell apoptosis in human proximal tubule cells [38,39]. We have previously demonstrated that zinc inhibits prostate BPH and PC-3 cells growth mainly by cell apoptosis through the mitochondrial pathway [7]. Now with the recognition of the zinc effect on MT 3 in BPH cells, it is possible that the role of MT 3 as a growth inhibitory factor may also involve in zinc-induced cell death in BPH cells.

Using human prostate cell models, we demonstrated that zinc treatment, at least in *in vitro *cell culture system, can restore the cellular zinc especially in the malignant cells, in which the endogenous zinc was depleted possibly due to the down-regulated MT expression through the MTF-1 involved mechanism. In turn, the restored zinc subsequently enhances MT gene expression significantly to secure the physiological and/or pharmacological level of zinc which will be recruited quickly by the proteins and enzymes to resume their bioactivities and eventually to control cell proliferation and/or death. Hence, zinc supplement may serve as a potential approach to modulate the molecular environment in prostate BPH and malignant cells aiming to enhance their responses to other chemotherapeutical treatment.

## Conclusion

This study provides important new information on zinc regulation of MT, isoforms 1, 2 and 3, expression in human prostate cells and tissues. Evidently, MT1/2 expression is significantly down-regulated in BPH, PC-3 cells, and human prostate malignant tissues; and MT 3 expression is a specific characteristic of BPH cells. Zinc restoration leads to re-establishing cellular homeostasis of zinc through up-regulation of MT gene expression, which may eventually result in resuming the critical selection for proliferation or death of the cells. The results suggest MT as a candidate tumor marker for diagnosis, prognosis and management guidance; and warrant further investigation of zinc as a potential supplement to benefit the BPH and prostate cancer treatment.

## Methods

### Human prostate cell lines and zinc treatment

Three human prostatic cell lines were used in this study: (a) HPR-1, a cell line derived from normal human prostate epithelial cells (kindly provided by Dr. C. K. Choo, University of Hong Kong, Hong Kong, China); (b) BPH, a cell line derived from human benign prostatic hyperplasia (BPH) tissue (kindly provided by Dr. S. Haywood, University of California, San Francisco); and (c) PC-3, a human malignant prostate cell line (ATCC, Rockville, MD). HPR-1 cells were cultured in keratinocyte medium supplemented with epidermal growth factor (EGF) (2.5 mg/500 ml) and bovine pituitary extracts (25 mg/500 ml) (Gibco BRL, Life Technologies, Bethesda, MD). BPH and PC-3 cells were cultured in RPMI-1640 medium with 5% and 10% of fetal bovine serum (FBS), respectively. All mediums were supplemented with penicillin and streptomycin (1 U/ml, Invitrogen), and the cells were maintained at 37°C in a humidified incubator with 5% CO_2_. The passages of three cell lines were within the range of 5 – 40.

Once the cells grew up to 50–60% confluence of the culture, the growth mediums were replaced by fresh serum/supplement-free medium for 24 h to synchronize cell growth. The cells were then treated with or without zinc sulfate (15–20 μM) in fresh serum/supplement-free medium for 0, 1, 3, 6, 12, and 24 h, respectively.

### Determination of MT levels by Western blot analyses

Total cellular proteins from the experimental samples were extracted by radioimmuno- precipitation assay(RIPA) lysis buffer (Upstate, Lake Placid, NY) containing protease inhibitor (Roche Diagnostics Gmbh, Mannheim, Germany). The protein extracts (30 ug/lane) were applied to electrophoresis on an 18% SDS-polyacrylamide gel, and then, a transferred poly(vinylidene fluoride) (PVDF) membrane (Millipore, Billerica, MA) was blocked in 5% nonfat dry milk in phosphate buffered saline (PBS) containing 0.05% Tween-20 (Sigma, St. Louis, MO) (PBST) for 1 hr at room temperature followed by the hybridization to the primary rabbit polyclonal antibody against MT1/2 (kindly provided by Dr. P.C. Huang, JHU), MT 3 (polyclonal antibody, kindly provided by Dr. G.F. Hu, Harvard University, MA), and β-actin (Sigma, St. Louis, MO) used as an internal control, respectively, at 4°C overnight. After being washed, membranes were incubated with corresponding second antibodies and washed again. The specific binding signals were visualized by enhanced chemiluminescence's system (Millipore, Billerica, MA, for MT1/2 and Pierce, Rockford, IL for β-actin) according to manufacturer's instructions. The target bands were scanned and quantified with an LKB Ultra Scan XL laser densitometer (Image Quant, Molecular Dynamics, Sunnyvale, CA).

### Reverse transcription and polymerase chain reaction assay (RT-PCR) for MT 3

Total RNA of the cells collected from the experiments was extracted using Qiagen (Valencia, CA) RNeasy mini kit according to the manufacturer's protocol. The quality of RNA was determined by the ratio of 28S/18S ribosomal RNA and the ratio of 260/280-nm absorbance. Two microgram of total RNA were used for complementary deoxyribonucleic acid (cDNA) synthesis with reverse transcriptase (ABI, Foster City, CA) in a total of 20 μl reaction. Primer sequences used for PCR to detect MT 3 were 5'*CCGTTCACCGCCTCCAG *3' (upper) and 5'*CACCAGCCACACTTCACCACA *3' (lower). PCR for MT 3 was conducted with cDNA template (1.0 μl) mixed with 0.8 μl of 5 μM upstream primer, 0.8 μl of 5 μM downstream primer, 0.4 μl of 10 mM deoxyribonucleotide triphosphate (dNTP), 0.8 μl of 50 mM MgCl_2_, 2 μl 10× Taq reaction buffer, and 0.08 μl of 5 U platinum Taq (Invitrogen) in a total of 20 μl reaction. The protocol used for the thermal cycler (PTC-100 MJ Research, Watertown, MA) was 94°C for 4 min, followed by 94°C for 30 s, 68°C for 30 s, and 72°C for 30 s at a total of 40 cycles, and followed by a final extension at 72°C for 7 min. Glyceraldehydes-3-phosphate dehydrogenase (GAPDH) was used as an internal control to normalize the MT 3 transcript level. Primer sequences for GAPDH were 5'*CCACCCATGGCAAATTCCATGGCA *3' (upper) and 5' *TCTAGACGGCAGGTCAGGTCCACC *3' (lower). The reaction of the PCR for GAPDH was similar to that for MT 3, except less cDNA template (0.4 μl) was used. The protocol for the thermal cycler was 94°C for 4 min, followed by 94°C for 30 s, 55°C for 30 s, and 72°C for 30 s for a total of 35 cycles and followed by a final extension at 72°C for 7 min. Eight microliter of each sample was electrophoresed in 1.2% agarose gels containing ethidium bromide and the target DNA fragments were visualized and quantified using an LKB Ultra Scan XL laser densitometer (Image Quant, Molecular Dynamics, Sunnyvale, CA).

### Immunofluorescence staining of MT1/2

The cellular distribution of MT1/2 proteins was determined by immunofluorescence staining in HPR-1, BPH, and PC-3 cells. The cells were grown on the cover slips placed in a 12-well plate and the following procedures were conducted with the cover slips. The cells were fixed with 4% paraformaldehyde (USB, Cleveland, Ohio) and permeabilized with 0.25% Triton ×-100 (Sigma, St. Louis, MO), respectively. For each treatment the cells were incubated with the reagent for 10 min at room temperature and then washed three times (10 min/each) in PBS. After 1 h incubation with PBS (10% FBS) at room temperature, the cells were incubated with rabbit polyclonal anti-MT1/2 antibody (1:500 dilution) in PBS (2% FBS) at 4°C overnight. The samples were then washed for five times (5 min/each) in PBS containing 0.05% Tween-20 and 1% BSA (washing buffer). The goat anti-rabbit FLURO-conjugated secondary antibody (1:500 dilution) (Invitrogen, San Francisco, CA) was applied and followed by the same washing procedure used for the primary antibody hybridization. For the nuclear staining, the cells were then incubated with Hoechst 33258 (0.2 μg/ml, Molecular Probes, Eugene, OR) for 10 min at room temperature and then washed three times in washing buffer. Finally, the cells were mounted to the slides with aqueous antifade medium (Polyscience, Warrington, PA). The MT1/2 immunofluorescence staining of the cells was analyzed and photographed using a fluorescence microscope (Nikon eclipse E800).

### Determination of cellular zinc

Prostatic cells were grown in 75 cm^2 ^flasks up to 80% confluence of the culture. The cells were treated with or without zinc (15 μM) in fresh serum-free medium for 3 h. Before harvest, the cells were washed once with 1× PBS and then washed twice after the collection to remove extracellular zinc. The cells were resuspended in sucrose buffer (250 mM sucrose, 20 mM HEPES, pH 7.4) and homogenized on ice. The nuclei and cell membranes were separated by centrifugation at 800 g for 10 min. The supernatants were then centrifuged at 10,000 g for 5 min, and these supernatants were used as cytosol samples. The protein concentrations of the samples were measured by Bradford method. Thirty microliters of each sample (200 μg of protein) were placed in a 96-well plate and mixed with 60 μl of TSQ buffer, which was composed of 1.9 g of sodium acetate, 2.9 g of sodium barbital, 1.5 mg of TSQ (Molecular Probes, Eugene, OR) dissolved in 100 μl of warmed ethanol, then dH_2_O was added to 100 ml, pH 10. The fluorescence of zinc labeled by TSQ was detected by using a Microplate Reader (Fluoroskan Ascent, Labsystems, Life Sciences International Company, USA) with excitation of 360 and emission of 495.

### Immunohistochemistry (IHC) study in human prostatic tissues

Twenty-three prostatic adenocarcinoma cases from archival paraffin embedded tissue blocks obtained from Pathology Department, Medical University of South Carolina and approved by the Institutional Review Board were included in this study. Examination of hematoxylin and eosin (H&E) stained sections revealed adenocarcinomas in 18 cases, normal prostatic glands in 5 cases, and all cases (n = 23) harbored BPH glands. The slides were coded without identification related to the patients. Immunohistochemistry (IHC) was carried out with rabbit anti-human/rat MT1/2 antibody (1:125 dilution) in a Dako cytomation autostainer with a standard protocol for IHC. Briefly, the samples of prostate were deparaffinized with heating and xylene incubation. Antigen retrieval was done by heating in ethylene diamine tetraacetic acid (EDTA), PH 9.0 buffer (Lab vision, Fremomt, CA) at 99.3°C for 20 min, incubated in 3% hydrogen peroxide, blocked with 5% skimmed milk for 5 min, incubated with MT1/2 antibody at room temperature for 30 min, followed by incubation with Horseradish peroxidase-labeled goat anti-rabbit IgG secondary antibody in a dilution of 1:200 for 30 min. Color was developed by incubating samples with diaminobenzidine (DAB)+chromogin for 10 min followed by Dako DAB enhancer for 5 min. Hematoxylin was used as counter stain. The specific staining of MT1/2 in the sections was examined using BX 50 Olympus microscope and the photographs were taken with attached Olympus DP 70 camera operated with DP Controller 2.1.1.183 software (Olympus Corporation). Semi-quantitative scoring of immunoreactivity was represented as: "negative" denoted no positive MT1/2 stained cells found; "+" denoted less than 10% of MT1/2 positive cells; "++" denoted 10–50% of MT1/2 positive cells; "+++" denoted more than 50% of MT1/2 positive cells [40]. The Gleason grade correlated to each case was defined as WHO standard 1–3 scale [41].

## Competing interests

The author(s) declare that they have no competing interests.

## Authors' contributions

HW carried out the molecular and cellular studies and involved in preparations of the figures to draft the manuscript. MMD completed the IHC studies on human samples and the data analysis. SL provided RT products for MT3 study and the discussion of RT-PCR experiments. DX helped in performance of immunofluorescence staining experiments, and RBF involved in extensive discussions. PF contributes to conception of the study, collection of the data and preparation of the manuscript. All authors read and approved the final manuscript.
